# A classification method of stress in plants using unsupervised learning algorithm and chlorophyll fluorescence technology

**DOI:** 10.3389/fpls.2023.1202092

**Published:** 2023-10-23

**Authors:** Miao Lu, Pan Gao, Jin Hu, Junying Hou, Dong Wang

**Affiliations:** ^1^ College of Mechanical and Electronic Engineering, Northwest A&F University, Yangling, Shaanxi, China; ^2^ College of Information Engineering, Northwest A&F University, Yangling, Shaanxi, China; ^3^ Key Laboratory of Agricultural Internet of Things, Ministry of Agriculture and Rural Affairs, Yangling, Shaanxi, China

**Keywords:** low temperature, chlorophyll fluorescence parameters, PSI, Fuzzy C-means clustering algorithm, uniform manifold approximation and projection

## Abstract

**Introduction:**

Chilling injury is one of the most common meteorological disasters affecting cucumber production. For implementing remedial measures as soon as possible to minimize production loss, a timely and precise assessment of chilling injury is crucial.

**Methods:**

To evaluate the possibility of detecting cucumber chilling injury using chlorophyll fluorescence (ChlF) technology, we investigated the continuous changes in ChlF parameters under various low-temperature conditions and created the criteria for evaluating chilling injury. The ChlF induction curves were first collected before low-temperature as unstressed samples and daily 1 to 5 days after low-temperature as chilling injury samples. Principal component analysis was employed to investigate the public information on ChlF parameters and evaluate the differences between samples with different degrees of chilling injury. The parameters (*F*_v_/*F*_m_, Y(NO), qP, and *F*_o_) accounted for a large proportion in the principal components and could characterize chilling injury. Uniform manifold approximation and projection method was employed to extract new features (Feature 1, Feature 2, Feature 3, and Feature 4) from ChlF parameters for subsequent classification model. Taking four features as input, a classification model based on the Fuzzy C-means clustering algorithm was constructed in order to identify the chilling injury classes of cucumber seedlings. The cucumber seedlings with different chilling injury classes were analyzed for ChlF images, rapid light curves, and malondialdehyde content.

**Results and discussion:**

The results demonstrated that the variations in these indicators among the different chilling injury classes supported the validity of the classification model. Our findings provide a better understanding of the relationship between ChlF parameters and the impact of low-temperature treatment on cucumber seedlings. This finding offers an additional perspective that can be used to evaluate the responses and damage that plants experience under stress.

## 1 Introduction

The frequency and severity of extremely low temperatures have gradually increased because of climate change (Xiao et al., 2021). Cucumber, as a typical cold-sensitive crop, is prone to low-temperature stress (Shibaeva et al., 2019). As one of the natural disasters that have the greatest impact on agriculture, low temperature limits the productivity and geographic distribution of cucumbers and has a serious impact on the world’s cucumber production and economic development (An et al., 2017). To minimize the output loss, a timely and accurate assessment of chilling injury is crucial for implementing remedial measures.

Low temperatures disrupt cellular structures and impair numerous vital physiological processes in plants, including the phase change of the chloroplast thylakoid membrane, by inactivating biological enzymes and causing the accretion of deleterious lipid peroxides (Barber and Andersson, 1992; Miura and Furumoto, 2013). However, due to the onerous and time-consuming operation steps, the aforementioned physiological and biochemical parameters might not be appropriate for non-destructive evaluation indicators of chilling injury in actual agricultural production settings. Photosynthesis is one of the core processes in plant life activities (Li et al., 2020). Nearly all aspects of photosynthetic activity are impacted by low temperatures, including photosystem II (PSII), photosystem I (PSI), cycle electron flow (CEF), and carbon fixation (Lu et al., 2020). PSI and PSII photoinhibition in cucumber leaves typically occur in environments with low temperature and normal light. Chilling stress tends to impede photosynthesis in the leaves, and excess light energy acquired by leaves will cause the formation of oxygen free radicals in the cells. The cell structure of the leaves would be damaged, resulting in yellowing of leaves, leaf curling, and even death, if the oxygen free radicals are not rapidly and effectively eliminated (Meng et al., 2008). The parameters related to photosynthesis could offer an excellent opportunity for the investigation of chilling injury.

Chlorophyll fluorescence (ChlF) technology, rapid and non-destructive, has been mentioned in previous studies as a method to assess plant damage caused by stress conditions and provide immediate feedback on the primary photochemistry processes (Moya et al., 2019). The ChlF induction curve could be used to evaluate the physiological state of PSII and other components of the photosynthetic electron transport chain. In a broader sense, ChlF parameters derived from induction curves are, directly or indirectly, related to all stages of photosynthetic reactions (Kalaji et al., 2016). Most studies examined the variations of ChlF parameters under low-temperature stress and chose one or more ChlF parameters susceptible to chilling injury for variety selection and analysis. The maximum photochemistry quantum yield of PSII (*F*_v_/*F*_m_), decreasing with increasing stress, could be a valuable and general indicator to characterize stress damage (Kumari et al., 2017; Aazami et al., 2021). Non-photochemical quenching (NPQ) mechanism, dissipating excessive excitation pressure accumulated in PSII reaction centers without causing adverse effects, could be used to analyze the plant photoprotection process (Kanazawa and Kramer, 2002). Additional ChlF parameters, such as effective quantum yield of PSII [Y(II)], photochemical quenching (qP), non-regulatory quantum yield of energy dissipation [Y(NO)], light-adapted steady-state fluorescence (*F*), and photochemical quenching coefficient (qL) were applied to explain and evaluate stress damage (Zhang et al., 2014). According to these findings, the parameters of the ChlF induction curve could be used to characterize chilling injury. However, utilizing a single or a combination of parameters to assess chilling injury has limitations because the parameters involve a number of intricate physiological and biochemical reaction processes in plants. It might be appropriate to employ a multi-index comprehensive evaluation method.

Most studies analyzed the degree of plant chilling injury in a supervised learning manner using ChlF technology, and the evaluation criteria relied heavily on the prior knowledge of human specialists (Dong et al., 2019). Prior knowledge refers to the reduction of temperature and the extension of duration, but it is often subjective. A promising solution to address the dearth of expertise in this sector is using unsupervised learning to classify plant stress injuries (Cao et al., 2015). Unsupervised learning, easy operation and strong applicability, do not require prior knowledge (Pu et al., 2021). But prior researches mainly employed this method to evaluate plant germplasm resources or annotate large-scale omics data sets for model plants (Henao-Rojas et al., 2021; Yan and Wang, 2022), and it was rarely used to evaluate the degree of plant damage under stress conditions.

**Figure 1:**
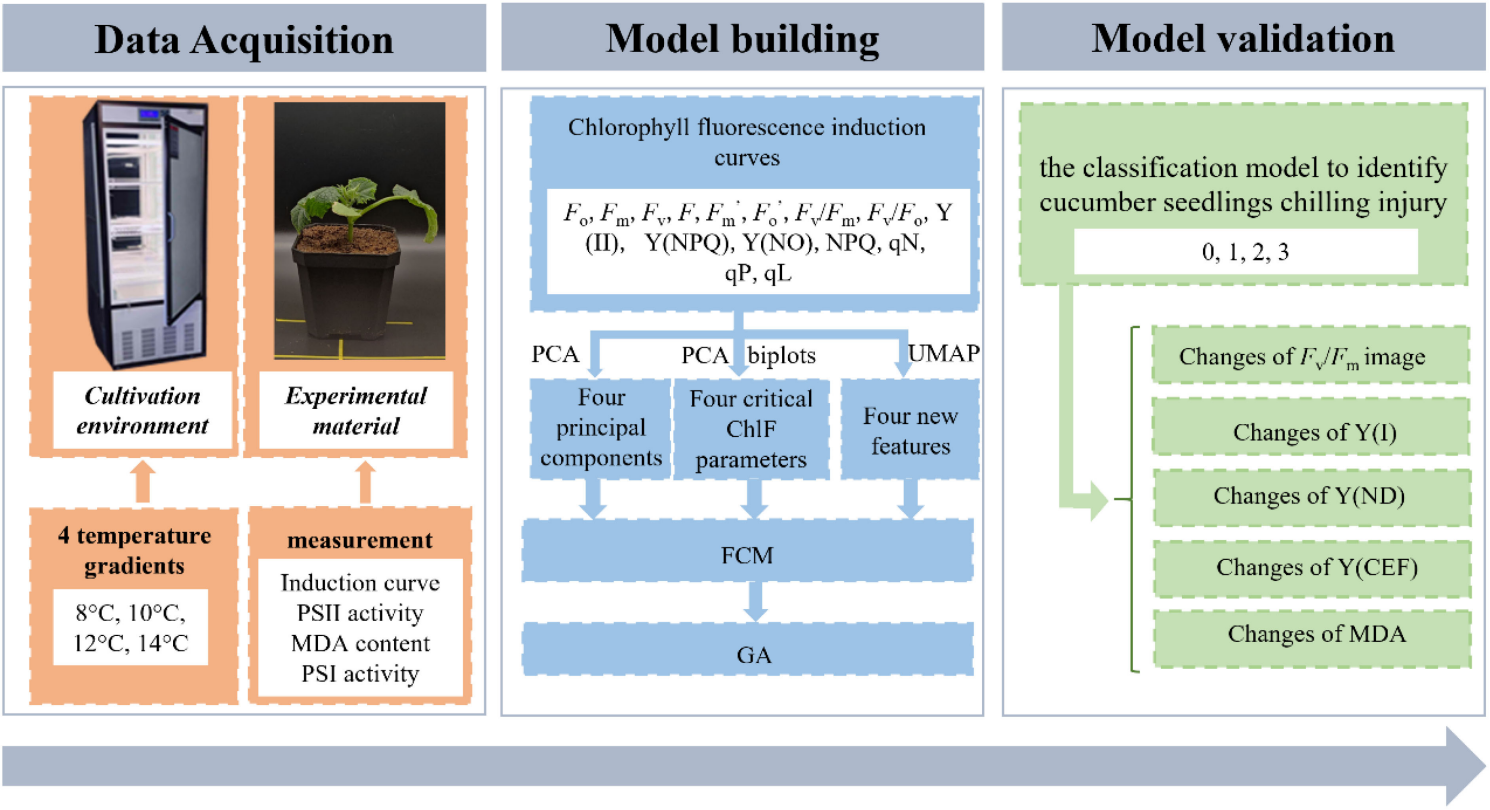
Flowchart of analysis and detection of chilling injury in cucumber seedlings leaves.

Considering that the precise classification of chilling injury is useful for subsequent disaster prevention and mitigation work, this study aimed to evaluate the potential of ChlF techniques in diagnosing plant chilling injury based on unsupervised learning methods. To this end, the specific objectives were to (1) analyze the changes in ChlF induction curve parameters under low-temperature treatment and identify available variables using the dimension reduction method; (2) create an unsupervised classification model using extracted variables as input; (3) validate the classification model and discuss the distinctions between samples of different chilling injury classes. The flowchart of typical steps for analyzing and detecting chilling injury proposed in this paper is illustrated in **Figure 1**.

## 2 Materials and methods

### 2.1 Experimental materials and methods

#### 2.1.1 Experimental materials and growth conditions

This experiment was carried out at the Key Laboratory of Agricultural Internet of Things, Ministry of Agriculture and Rural Affairs, Northwest A&F University, from December 2020 to January 2021. Cucumber (*Cucumis sativus* L. cv Bonai 14-3) was germinated and grown in 72-hole seed trays. The seedlings were transferred to plastic nutrition cups (10×10×9 cm^3^) with the Danish substrates (Pindstrup, Denmark) when they had two leaves and one heart. An artificial climatic chamber (RGL-P500D, Hefei) with three vertical full spectrum LEDs and an external ultrasonic humidifier provided a growing environment for the seedlings. The environmental settings in the chamber were as follows: the light intensity was 140 μmol·m^-2^·s^-1^ and the period was 14 h/10 h (light/dark). The air temperature during the light and dark were 25°C and 16°C, respectively. The relative humidity during the light and dark were 60% and 50%, respectively. The vapor pressure deficit during the light and dark were 1.1~1.5 kPa and 0.4~0.5 kPa, respectively. After seven days of acclimatization, the cucumber seedlings were separated into four groups with 24 cups each. Different groups of cucumber seedlings were placed in four different climate chambers. The air temperature of the four different climate chambers were 8°C, 10°C, 12°C, and 14°C, respectively. Other environmental parameters were consistent with the acclimatization period.

All plants were exposed to a low-temperature environment for five days. Subsequently, to evaluate their recovery, the two groups of cucumber seedlings at 8°C and 14°C were transferred to their previous temperature treatment (light/dark: 25°C/16°C) for 5 days.

Eight plants were randomly chosen as samples from each group to measure the ChlF induction curve. Four plants were randomly chosen as samples to measure the ChlF image and the activity and energy conversion of PSI and PSII. Twelve plants were selected for malondialdehyde (MDA) content measurement after each day of low-temperature light exposure.

#### 2.1.2 Measurement of the ChlF parameters

The measurements were performed between 3 h and 6 h after the beginning of the photoperiod (10:00 h) every day. Eight plants were randomly selected as the samples from each group to measure the ChlF induction curve, and the second fully unfolded leaf from the growth point down was taken as the leaf to be tested. Cucumber seedlings were dark-adapted for over 20 mins prior to measurement. MINI-PAM-II measuring systems (Heinz Walz, Effeltrich, Germany) were utilized to measure the ChlF induction curve. *F*_o_ and *F*_m_ were determined with a very weak light (470nm,<0.1 μmol·m^-2^·s^-1^) and a transient saturated light pulse (470nm, >3000 μmol·m^-2^·s^-1^), respectively (Chen et al., 2021). The samples were then illuminated for 300 s with a continuous actinic light (900 μmol·m^−2^·s^−1^) to obtain a steady-state fluorescence yield (*F*). Subsequently, saturating light pulses were applied to achieve $F_{\text{m}}^{,}$ . Actinic light was then turned off and a far-red illumination was turned on to measure $F_{\text{o}}^{,}$ (Lima et al., 2002). Other ChlF parameters in the induction curve are displayed in **Table 1**. Four plants were randomly chosen as samples to measure the ChlF false-color image and the activity and energy conversion of PSI and PSII, and the second fully unfolded leaf from the growth point down was taken as the leaf to be tested. A blue version of IMAGING-PAM-MAX/B measuring systems (Heinz Walz, Effeltrich, Germany) was utilized to collect the images. The false-color images (*F*_v_/*F*_o_, *F*_o,_ and *F*_m_) were measured using the standard measurement procedure of the ImagingWin software (Dong et al., 2019) and created by mapping the one-dimensional value to the three-dimensional RGB value using a false-color system. DUAL-PAM-100 measuring systems (Heinz Walz, Effeltrich, Germany) were utilized to measure the rapid light response curves (RLCs) using the standard measurement procedure of the Dual-PAM-100 software (Yang et al., 2020). A saturation pulse after illumination with far-red light for 10 s was used to measure the Pm after dark-adaption. The gradients of light intensity were: 0, 8, 34, 92, 170, 270, 419, 609, 921, 1453, and 2256 μmol·m^-2^·s^-1^. The parameters used in this paper are displayed in **Table 1**.

**Table 1:** the definition and description of the parameters.

Parameter	Definition	Description
*F*	/	the light-adapted steady-state fluorescence
*F*_o_	/	minimum ChlF
*F*_m_	/	maximum ChlF
$F_{\text{o}}^{,}$	/	minimum ChlF after light adaptation
$F_{\text{m}}^{,}$	/	maximum ChlF after light adaptation
*F*_v_	*F*_m_−*F*_o_	variable ChlF
*F*_v_/*F*_m_	(*F*_m_−*F*_o_)/*F*_m_	maximum photochemistry quantum yield of PSII
*F*_v_/*F*_o_	(*F*_m_−*F*_o_)/*F*_o_	the potential activity of PSII
Y(II)	( $F_{\text{m}}^{,}$ −F)/ $F_{\text{m}}^{,}$	practical photochemistry quantum yield of PSII
Y(NO)	*F*/*F*_m_	unregulated heat dissipation and quantum yield of fluorescence emission
Y(NPQ)	1−Y(II)−Y(NO)	non-photochemical fluorescence quenching quantum yield under light induction
NPQ	(*F*_m_− $F_{\text{m}}^{,}$ )/ $F_{\text{m}}^{,}$	non-photochemical fluorescence quenching
qL	$\text{qP}\times F_{\text{o}}^{,}/F$	photochemical quenching coefficient
qP	( $F_{\text{m}}^{,}$ −*F*)/( $F_{\text{m}}^{,}-F_{\text{o}}^{,}$ )	photochemical quenching coefficient
qN	1−( $F_{\text{m}}^{,}-F_{\text{o}}^{,}$ )/(*F*_m_−*F*_o_)	non-photochemical quenching coefficient
*P*	/	P700 real-time signal under actinic light
*P*_m_	/	Maximum P700+ signal
$P_{\text{m}}^{,}$	/	Maximum P700+ signal after light adaptation
Y(I)	( $P_{\text{m}}^{,}$ −*P*)/*P*_m_	the quantum yield of PSI
Y(ND)	*P*/*P*_m_	the P700 oxidation ratio in a given actinic light
ETR(I)	Y(I)×PAR×0.84×0.5	the electron transfer rate of PSI
ETR(II)	Y(II)×PAR×0.84×0.5	the electron transfer rate of PSII
Y(CEF)	ETR(I)−ETR(II)	the cyclic electron flow value between PSI and PSII

/, indicates that there are no definition for these parameters.

#### 2.1.3 Measurement of Malondialdehyde content

Twelve plants were selected for malondialdehyde (MDA) content measurement after each day of low-temperature light exposure. The thiobarbituric acid (TBA) colorimetry method was employed (Xu et al., 1993; Ruan et al., 2002). For each plant, 0.5 g fresh leaf material was homogenized with 5 ml of 10% trichloroacetic acid (TCA). 2 ml of extract solution was combined with 2 ml of 0.6% TBA solution and heated in a boiling water bath for 15 mins and then cooled and centrifuged for 10 mins. A UV spectrophotometer (LAMBDA 365, America) was used to measure the absorbance of the reaction mixture at 450 nm, 510 nm, 532 nm, and 560 nm, respectively. The MDA content was calculated according to the following formula.


(1) $$C=\left\{6.45\left[D_{532}-\left(D_{510}-D_{560}\right)/2\right]-0.56D_{450}\right\}\times \frac{N}{W}$$

Where C represents the MDA content, in nmol·g^-1^. $D_{450}$ , $D_{510}$ , $D_{532}$ , $D_{560}$ represent the absorbance values at wavelengths of 450 nm, 510 nm, 532 nm, and 560 nm, respectively. *N* represents the volume of extraction liquid. *W* represents the fresh plant tissue weight.

### 2.2 Modeling approaches

The dataset comprised 15 ChlF parameters obtained from the induction curve (*F*_o_, *F*_m_, *F*_v_, *F*, $F_{\text{m}}^{,}$ , $F_{\text{o}}^{,}$ , *F*_v_/*F*_m_, *F*_v_/*F*_o_, Y(II), Y(NPQ), Y(NO), NPQ, qN, qP, qL). Pearson correlation analysis was used to explain the association between different parameters. Principal component analysis (PCA) and uniform manifold approximation and projection (UMAP) were used to extract critical information from the dataset. The fuzzy C-means clustering algorithm (FCM) and genetic algorithm (GA) were used to build a classification model. All calculations were performed with at least three independent biological replicates.

#### 2.2.1 Data normalization

To facilitate subsequent data analysis, the data set was normalized in the range [0,1] using a linear normalization function defined by:


(2) $$Y=\frac{X-X_{\min}}{X_{\max}-X_{\min}}$$

Where *X*, *Y* represents the data to be normalized and normalized, and *X*_min_, *X*_max_ represent the minimum and maximum in the data sequence. Meanwhile, we apply the 3σ rule to filter the data for outliers (Lehmann, 2013).

#### 2.2.2 Data analysis

Pearson correlation coefficient is an index that accurately measures the correlation between two variables. In this study, it could be used to examine the relationship between different ChlF parameters. For two different ChlF variables $A=\left[a_{1},a_{2},\ldots ,a_{n}\right]$ and $B=\left[b_{1},b_{2},\ldots ,b_{n}\right]$ , the formula was given by (Wiedermann and Hagmann, 2016):


(3) $$P_{A,B}=\frac{n\sum AB-\sum A\sum B}{\sqrt{n\sum A^{2}-{\left(\sum B\right)}^{2}}\sqrt{n\sum B^{2}-{\left(\sum B\right)}^{2}}}$$

The value range of $P_{A,B}$ is [-1,1], that is $\left|P_{A,B}\right|=\left[0,1\right]$ . The correlation between *A* and *B* is judged by the following range of values: $\left|P_{A,B}\right|$ is extremely strong at 0.8 and 1.0, strong at 0.6 and 0.8, moderate at 0.4 and 0.6, weak at 0.2 and 0.4, and uncorrelated at 0.0 and 0.2.

Statistical analyses of the ChlF parameters were conducted using Levene’s test and the one-way ANOVA test in SPSS (SPSS Inc., Chicago, IL). The Levene’s test can be employed for variance homogeneity. If its p-value is less than 0.05, then we can reject the null hypothesis of variance homogeneity and conclude that there is significant heterogeneity among the groups. The one-way ANOVA test was performed to examine the differences in means among the groups. The F-value obtained from the ANOVA test represents the ratio of between-group variability to within-group variability. A larger F-value suggests a greater difference in means among the groups, indicating a significant effect. Conversely, a smaller F-value indicates a smaller difference in means, suggesting a non-significant effect.

#### 2.2.3 Dimension reduction

The PCA method is primarily used to decrease the number of variables and discover the relationship structure between similarly classified variables. In doing so, the main input is transformed into principal components (PCs) that are non-correlated. Most of the variations in the original data may be explained by fewer indicators (Dittrich et al., 2021). The percentage of variance in the data that the PCs could explain was set at 95% (Sun et al., 2021).

Uniform manifold approximation and projection (UMAP) was proposed in 2018 to reduce dimensionality and improve visualization (Mcinnes and Healy, 2018). Based on Riemannian geometry and algebraic topology, UMAP not only has the speed advantages of PCA but also retains the local and global structure. The hyper-parameters of UMAP were set as follows: The dimensionality of the target embedding, n = 4; the number of neighbors, k = 5; the minimum allowed distance between points in the embedding space, d = 0. In this study, PCA and UMAP were applied to process ChlF parameters and select the most important variables to assess chilling injury in cucumber seedlings.

#### 2.2.4 Clustering algorithm

The fuzzy C-means clustering algorithm (FCM) is an improvement of the conventional K-means algorithm. When used in conjunction with fuzzy theory, FCM calculates the similarity between the samples and the cluster centers and estimates the probability that a sample fits into a particular category according to the membership (Kamolov and Park, 2021). The FCM algorithm iteratively optimizes the objective function *J* by updating the membership *u_ij_* and the cluster center *V*. The objective function *J* could be expressed as:


(4) $$J=\sum_{i=1}^{N}\sum_{j=1}^{C}u_{ij}^{m}d_{ij}^{2}$$

Where *m* represents the fuzzy coefficient, *m*>1. *N* represents the sample size. *d_ij_* represents the Euclidean distance from the sample to cluster center.

The traditional FCM randomly selects a sample as the initial centroid, however, this may cause the model to fall into a local optimum. Genetic algorithm (GA) is a popular optimization algorithm inspired by the idea of biological evolution that generates high-quality optimal solutions based on selection, crossover, and mutation processes (Reddy et al., 2020). Thus, the initial centroid of FCM was optimized by GA in the proposed chilling injury classification model. In the optimization process, genetic parameters were set as follows: population 100, crossover probability 0.8, mutation probability 0.2. Taking selection into account, individuals were chosen based on the Euclidean distance between the sample set and the chromosome of the population.

## 3 Results and discussions

### 3.1 Changes of ChlF parameters under experimental conditions

**Figure 2:**
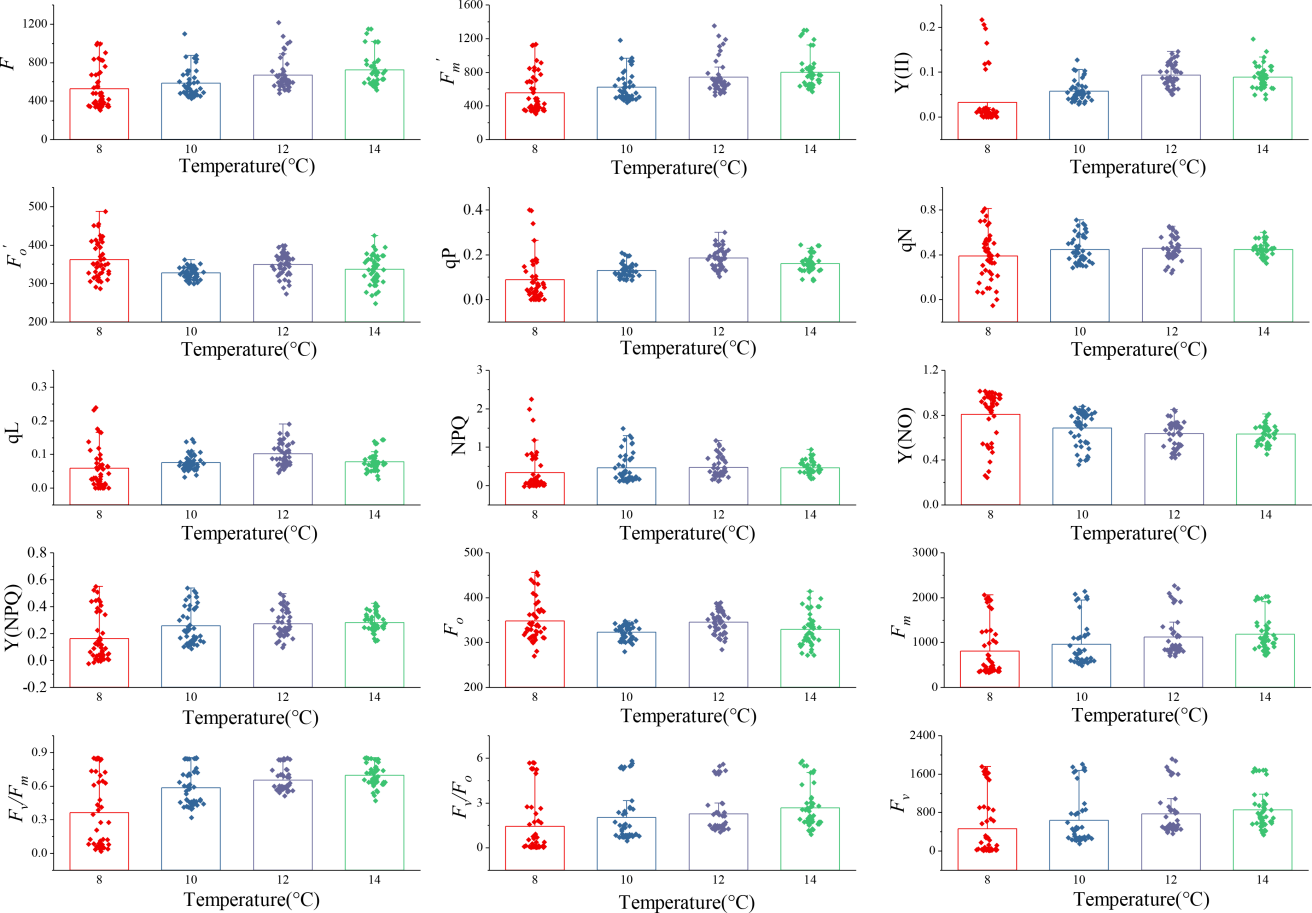
Changes of ChlF parameters with 0 to 5 days of four low-temperature treatments. The parameters were *F*, $F_{\text{m}}^{,}$ , Y(II), $F_{\text{o}}^{,}$ , qP, qN, qL, NPQ, Y(NO), Y(NPQ), *F*_o_, *F*_m_, *F*_v_/*F*_m_, *F*_v_/*F*_o_, and *F*_v_, respectively. Each subplot represented a ChlF parameter. Each column in the subplot represents the distribution of the ChlF parameter with 0 to 5 days at one temperature, n=48. Each point was the average value obtained from three repetitions of the experiment.

Fifteen ChlF parameters were obtained from the induction curve measured using the MINI-PAM-II measurement system. Changes in ChlF parameters at four low-temperature treatments over a period of 0~5 days are shown in **Figure 2**; **Table 2**. Under different temperature treatments, the ChlF parameters had large intergroup differences and exhibited heteroscedasticity. Some ChlF parameters, such as *F*_m_, *F*_v_, *F*, *F*_v_/*F*_m_, *F*_v_/*F*_o_, $F_{\text{m}}^{,}$ , and Y(NPQ), changed in the same order as the experimental conditions. Additionally, *F*_o_, Y(NO), and $F_{\text{o}}^{,}$ negatively correlated, while other ChlF parameters were positively correlated, with the experimental temperature. The distribution of ChlF parameters at 12°C and 14°C treatment was comparable, and both showed more minor distribution disparities, whereas more significant distribution changes were observed at 8°C and 10°C treatments. Particularly during the 8°C treatment, there were more extreme ChlF parameter values. The possible causation was that 8°C is past the threshold temperature that cucumber seedlings could withstand, and the PSII of cucumber leaves was irreversibly damaged (Anwar et al., 2018). The different and regular variations in the trends of the ChlF parameters during low-temperature treatment indicated using them as model input could be appropriate for evaluating chilling injury.

**Table 2:** Changes of ChlF parameters with 0 to 5 days of four low-temperature treatments.

ChlF parameters	Temperature (°C)								p	F
	8		10		12		14			
	Mean	Std	Mean	Std	Mean	Std	Mean	Std		
*F*	528.6	206.5	583.6	145.4	668.7	166.3	723.2	169.4	0.183	12.008
$F_{\text{m}}^{,}$	554.6	237.0	622.3	167.9	739.8	195.3	796.8	201.1	0.188	14.200
Y(II)	0.033	0.060	0.058	0.023	0.093	0.025	0.089	0.026	0.186	28.736
$F_{\text{o}}^{,}$	361.9	49.0	327.0	14.8	349.1	28.9	337.2	38.3	0.000	8.599
qP	0.087	0.098	0.129	0.031	0.185	0.045	0.159	0.039	0.000	19.143
qN	0.386	0.211	0.446	0.124	0.455	0.102	0.446	0.066	0.000	2.846
qL	0.059	0.060	0.076	0.024	0.101	0.034	0.078	0.027	0.000	6.001
NPQ	0.338	0.531	0.457	0.381	0.475	0.270	0.466	0.174	0.056	1.611
Y(NO)	0.806	0.223	0.686	0.156	0.634	0.113	0.631	0.082	0.002	14.013
Y(NPQ)	0.161	0.173	0.256	0.137	0.272	0.099	0.281	0.068	0.000	9.604
*F*_o_	347.7	45.0	322.5	16.4	345.3	24.8	329.4	36.6	0.000	6.604
*F*_m_	805.9	581.6	960.1	518.4	1115.9	455.6	1183.5	386.3	0.372	5.727
*F*_v_/*F*_m_	0.361	0.316	0.584	0.161	0.652	0.102	0.694	0.097	0.000	29.719
*F*_v_/*F*_o_	1.433	1.971	2.015	1.721	2.262	1.414	2.673	1.381	0.507	4.879
*F*_v_	458.1	599.3	637.5	524.5	770.6	460.0	854.1	398.6	0.398	5.783

n = 48, p<0.05. Mean represents the average value of the ChlF parameter, std represents the standard deviation of the ChlF parameter.

**Figure 3:**
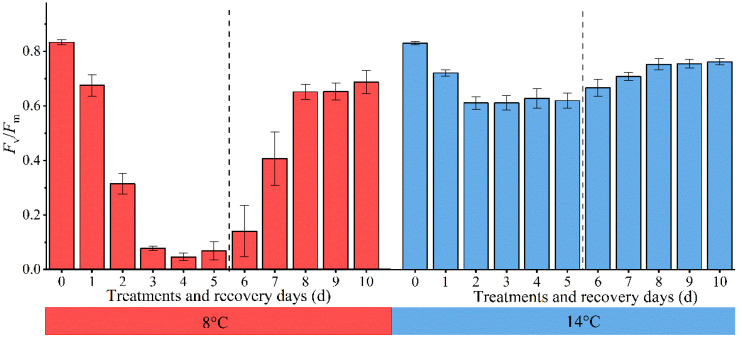
Changes of cucumber leaves *F*_v_/*F*_m_ under low temperature and subsequent recovery. The 0 d ~ 5 d in the abscissa represent low-temperature treatment, and the 6 d ~ 10 d represent suitable-temperature treatment.

The evaluation of chilling injury in cucumber seedling was related to their response to low temperatures and subsequent recovery at the appropriate temperature. *F*_v_/*F*_m_, as a general indicator to determine the condition of the photosynthetic apparatus and estimate stress damage in plants (Lin et al., 2021), changes regularly with the degree of chilling injury. We further evaluated the variations of cucumber leaves *F*_v_/*F*_m_ each day at 8°C and 14°C treatments and subsequent recovery (**Figure 3**). The values of *F*_v_/*F*_m_ decreased gradually with decreasing temperature and increasing duration. When cucumber seedlings were not exposed to low temperatures, the *F*_v_/*F*_m_ values remained stable at around 0.84. The *F*_v_/*F*_m_ values at 8°C were more rapidly decreased than those at 14°C in cucumber leaves. The *F*_v_/*F*_m_ values at 8°C decreased to approximately 0.1 in cucumber leaves treated for 5 days, whereas in those treated at 14°C, the *F*_v_/*F*_m_ value remained at about 0.6. There were significant differences between them, demonstrating that the photosynthetic apparatus suffered more severe damage at 8°C compared to 14°C and PSII is relatively stable at 14°C. Subsequently, when the cucumber seedlings were transferred to their previous temperature environment, the recovery rates of cucumber leaves’ *F*_v_/*F*_m_ values at 8°C were higher than those at 14°C. The 8°C and 14°C-treated cucumber seedlings *F*_v_/*F*_m_ reached 82.5% and 91.8% of their pre-low temperature treatment levels after 5 days of recovery. It has been suggested that PSII recovery system in the plants was not damaged and chilling injury had little effect on it when *F*_v_/*F*_m_ reached above 0.8 or 95% of their pre-low temperature treatment level (Ríos-Meléndez et al., 2021; Takeuchi et al., 2022). Thus, the chilling injury was reversible in this case and could be regarded as slight. Our results did not meet the above conditions, indicating that the effects of chilling injury were still present. Meanwhile, the samples’ response to different low-temperatures and subsequent recovery were different. On this account, such chilling injury could be regarded as moderate and severe. Therefore, the subsequent model could be configured with 4 centroids, each corresponding to an unstressed state, slight chilling injury, moderate chilling injury, and severe chilling injury, respectively. This scheme also referred to previous literature (Ali et al., 2014).

### 3.2 Classification model of chilling injury

#### 3.2.1 Pearson correlation analysis for the ChlF parameters

**Figure 4:**
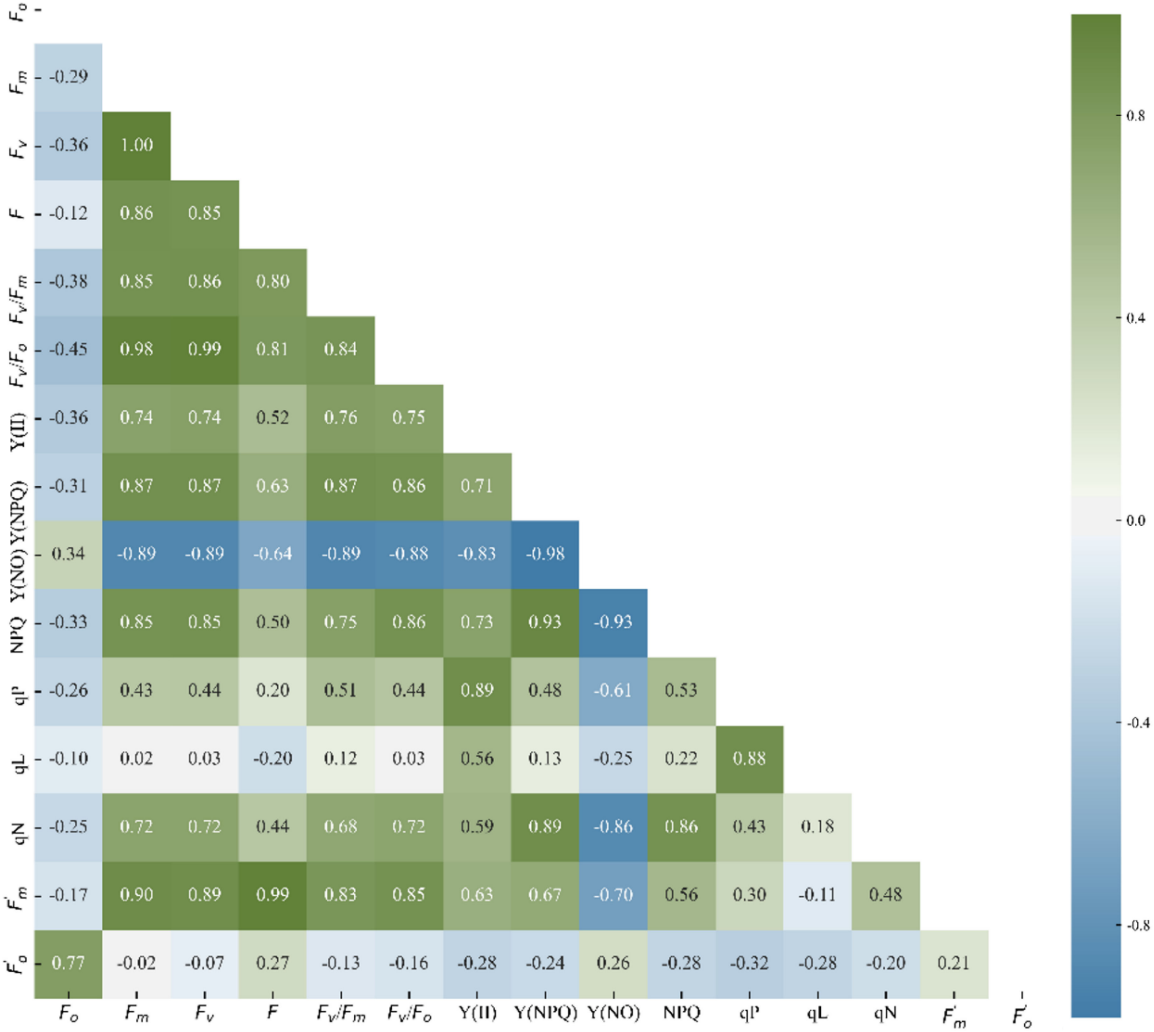
Correlation coefficients between ChlF parameters.

Analyzing the correlations among ChlF parameters provides a preliminary understanding of their mathematical relationships. **Figure 4** depicts the correlation coefficient between ChlF parameters under experimental conditions. Only data from the lower triangle was displayed, as the matrix is symmetric. *F*_v_/*F*_m_ is strongly positively correlated with *F*_v_/*F*_o_, *F*_v_, Y(II), $F_{\text{m}}^{,}$ , Y(NPQ), qN, NPQ, *F*_m_, *F*, but not with $F_{\text{o}}^{,}$ and qL. *F*_o_ was only strongly correlated with $F_{\text{o}}^{,}$ , but weakly or not correlated with other parameters. Y(NO) was extremely strongly correlated with many parameters, particularly Y(NPQ) and NPQ. There were large or small correlations between all the ChlF parameters, and each parameter had at least a significant or extremely significant correlation with another parameter. It may not be reasonable to manually select one or multiple parameters to characterize the chilling injury. If all ChlF parameters were employed for subsequent data analysis, data redundancy would occur, resulting in intensive calculation and strong data fluctuation. It is necessary to extract the effective components of ChlF parameters.

#### 3.2.2 Dimension reduction for the ChlF parameters

**Figure 5:**
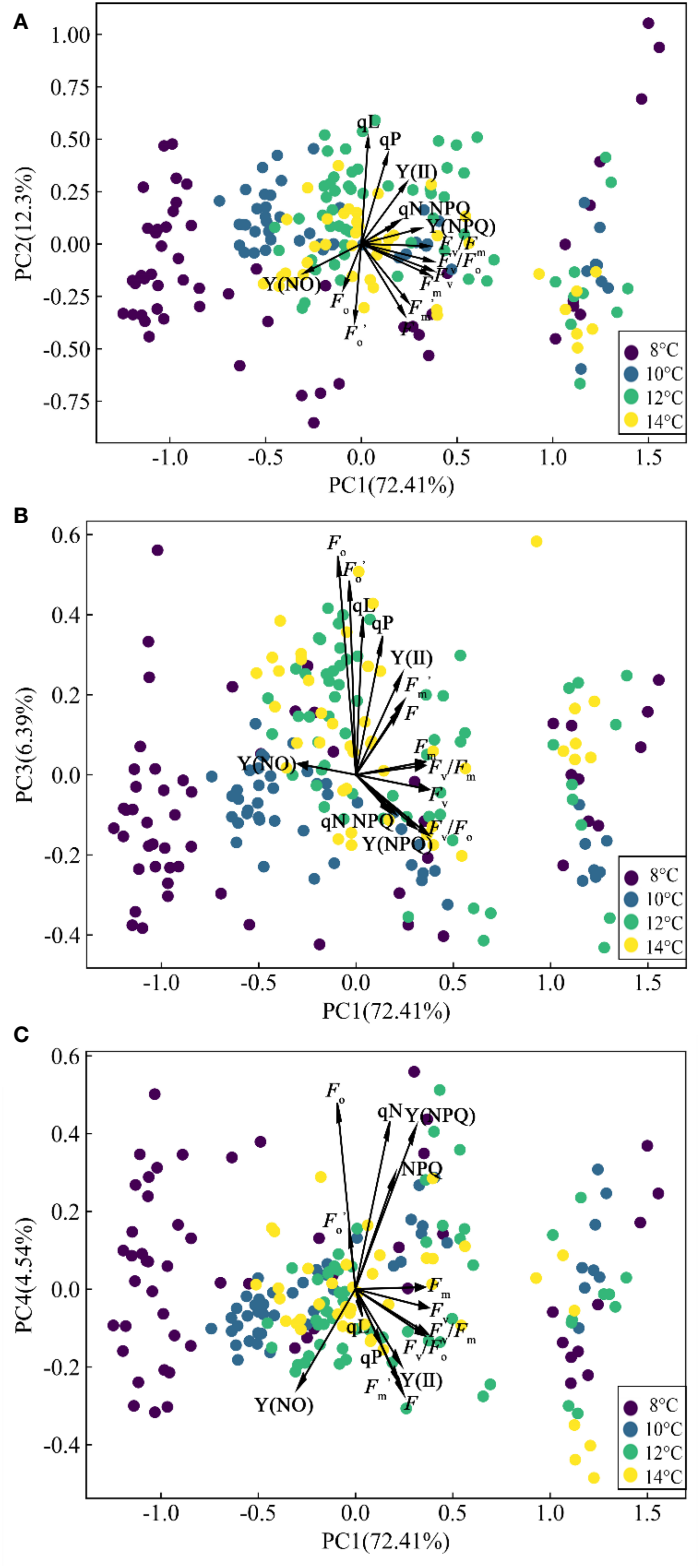
Biplot resulting from PCA. **(A)** PC1 and PC2. **(B)** PC1 and PC3. **(C)** PC1 and PC4.

All the clustering algorithms have limitations of dimensionality because high dimensional data requires more observed samples to produce much density (Allaoui et al., 2020). Methods of dimension reduction (PCA and UMAP) make it easier for these algorithms to cluster the data. Public information on ChlF parameters was extracted using the PCA method, and several comprehensive and independent indicators containing most of the original information were constructed (**Figure 5**).

The first four PCs accounted for 95.639% of the original information. The first axis (PC1) explained 72.413% of the variance and was dominated by the damage of PSII in cucumber leaves at low temperature. The parameters with a high positive contribution in PC1 are *F*_v_/*F*_m_, *F*_v_, *F*_v_/*F*_o_, and *F*_m_. These parameters were obtained before continuous actinic light. The damage of low temperature to photosynthetic organs can lead to significant changes in these parameters. The reduced photosynthesis in low temperature conditions can lead to the accumulation of excessive energy. Excessive excitation energy reached the reaction center and dissipated through the antenna chlorophyll, which caused a sharp decline in PSII activity, and caused extensive damage to the PSII complex (Zhang et al., 2020). Then, the second axis (PC2), which was related to the PSII activity in cucumber leaves as shown by the loadings of qP, qL, *F*, and $F_{\text{o}}^{,}$ , explained 12.297% of the variance. F and $F_{\text{o}}^{,}$ were negatively distributed on the PC2, while qP and qL were positively distributed. Low temperature treatment reduces the PSII activity and increases the risk of photoinhibition, resulting in a decrease in qP (the approximate fraction of open PSII reaction center) and an increase in NPQ. Thermal energy dissipation might not be sufficient to prevent a decline in qP at low temperatures, hence the negative correlation between qP and NPQ was manifested in PC3 (Kitao et al., 2004). The third axis (PC3) accounted for 6.389% of the variance and included the photoprotection ability of PSII in cucumber leaves. PC3 was dominated by *F*_o_ and Y(NPQ). Y(NPQ) indicates the degree of PSII photoprotection (Dias et al., 2018). Plants reduced the impact of low temperature stress by increasing the ratio of energy dissipation [Y(NPQ) and Y(NO)]. The fourth axis (PC4) accounted for 4.54% of the variance and was dominated by the efficiency of PSII in cucumber leaves, as it mainly integrated the information of Y(NO) and *F*_o_. In PC3 and PC4, *F*_o_ had the largest positive contribution. In PC1 and PC4, Y(NO) had the largest negative contribution. *F*_o_ is related to the chlorophyll concentration and the transfer efficiency of excitation energy (Georgieva and Lichtenthaler, 1999). Low temperature treatment reduces the energy transfer efficiency of antenna chlorophyll a to the PSII reaction center (inactive), which could explain the rise of *F*_o_. Considering the result of correlation analysis, *F*_v_/*F*_m_, qP, Y(NO), and *F*_o_ were selected as key ChlF parameters sensitive to chilling injury of cucumber seedlings. This finding is supported by the research conducted by Dong et al. (2019) and Aazami et al. (2021). The aforementioned analysis showed the distribution of different ChlF parameters in the PCA-extracted general information and indicated several fluorescence parameters that could better characterize chilling injury.

**Figure 6:**
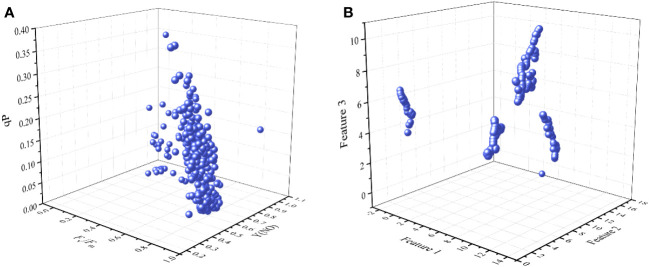
Visualization result. **(A)** the 3D scatter plot with *F*_v_/*F*_m_, Y(NO), and qP. **(A)** the 3D scatter plot with Feature 1, Feature 2, and Feature 3.

The four PCs and sensitive ChlF parameters were extracted by PCA, which may be important for early diagnosis of cucumber chilling injury. However, the loose distribution of data transformed by PCA may compromise the precision of subsequent clustering models (**Figure 5**). Similarly, we analyzed the distribution of ChlF parameters [*F*_v_/*F*_m_, Y(NO), and qP] that contributed significantly to the PCs, but it has the same shortcomings as PCA (**Figure 6A**). UMAP seemed more appropriate for dimension reduction. Compared with PCA, the visualization result was superior when we applied the UMAP (**Figure 6B**). All samples were reduced to four dimensions using UMAP, and the new features of the samples were Feature 1, Feature 2, Feature 3, and Feature 4, respectively. It should be highlighted that UMAP successfully reflects much of the large-scale global structure that is well represented by PCA while also preserving the local fine structure. However, UMAP lacks the robust interpretability of PCA. As a result, we applied PCA to explain the significance of various ChlF parameters in chilling injury and used UMAP to reduce the sample dimensionality.

#### 3.2.3 Classification model for cucumber seedlings chilling injury

**Figure 7:**
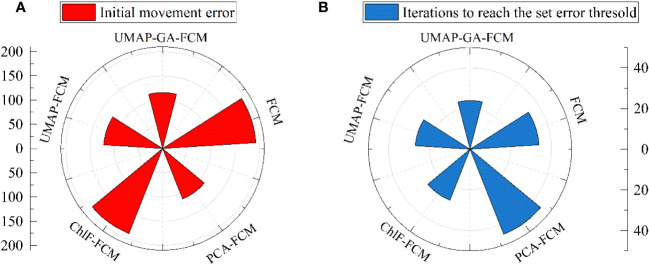
Comparison of results between different methods. **(A)** the initial movement errors. **(B)** the iterations to reach the set error threshold.

Different models, including FCM (15 ChlF parameters as input), ChlF-FCM (*F*_v_/*F*_m_, *F*_o_, Y(NO), and qP as input), PCA-FCM (4 PCs as input), and UMAP-GA-FCM (Feature 1, Feature 2, Feature 3, and Feature 4 as input), were constructed for comparison and selection (**Figure 7**). Using the GA algorithm to optimize the first centroid can reduce the initial movement error and the iterations to a certain extent. Significant differences were observed in the initial movement error of ChlF-FCM and FCM, but they did not require a lot of iterations. The PCA, as a linear method, has the tendency to neglect some important local information. If all ChlF parameters for the datasets in this paper were used, it would result in a dimensional disaster. Correspondingly, the outcomes of models that used them as inputs were not the best. All models can ultimately reach the set error threshold, but the UMAP-GA-FCM method has the smallest initial error and iteration steps. Therefore, the UMAP-GA-FCM method was employed to evaluate the chilling injury in this study.

**Figure 8:**
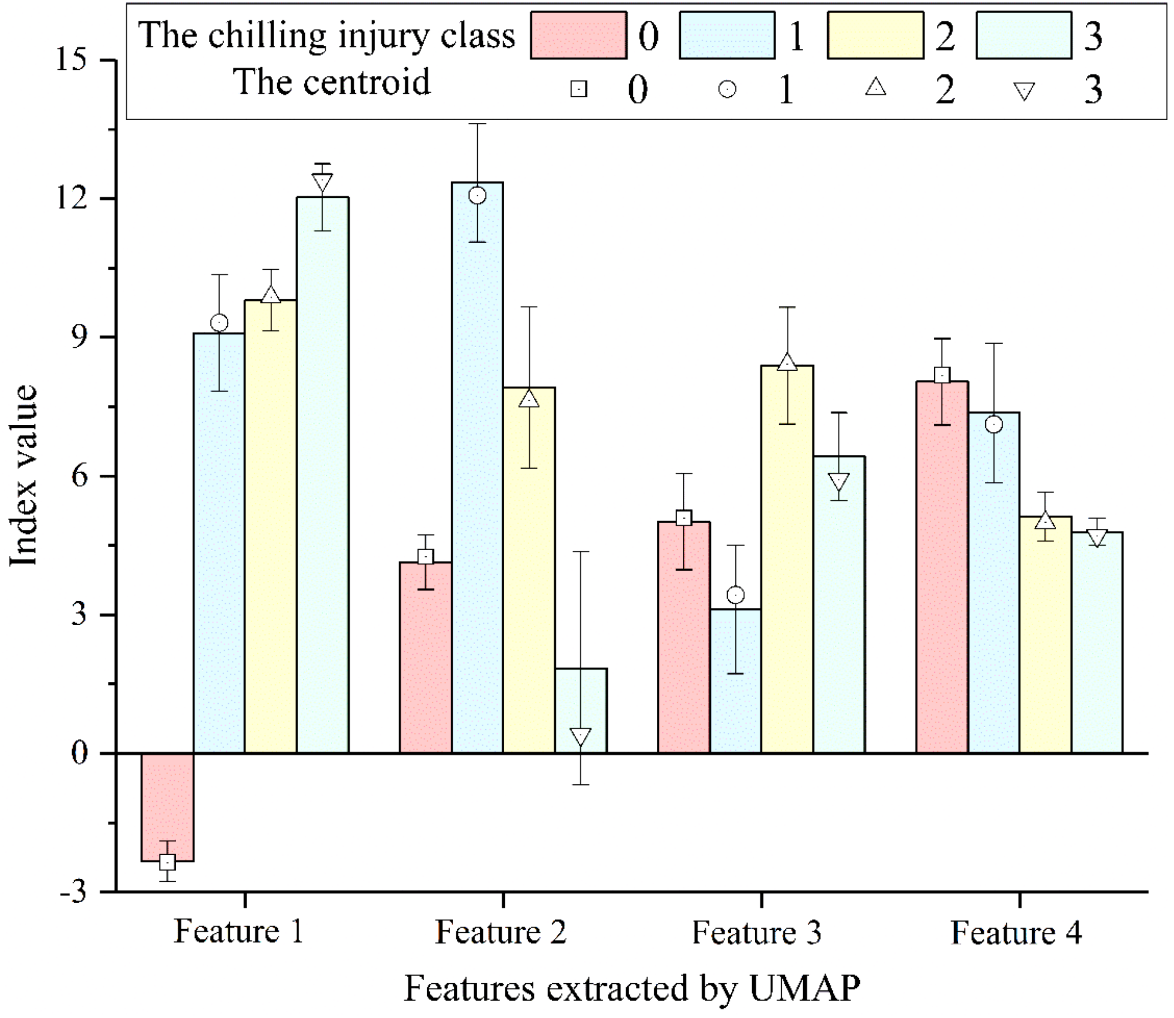
The corresponding relationship between the new features and centroids.

Taking the four new features obtained by the UMAP method as the input, an evaluation model of cucumber seedlings chilling injury was constructed based on the UMAP-GA-FCM algorithm. The corresponding relationship between the new features and centroids is illustrated in **Figure 8**. The UMAP-GA-FCM algorithm was successful at pulling together clusters corresponding to similar samples. Samples from different classes exhibit distinct feature values and centroids, while the centroids and the average values of the features at different classes were generally consistent.

**Figure 9:**
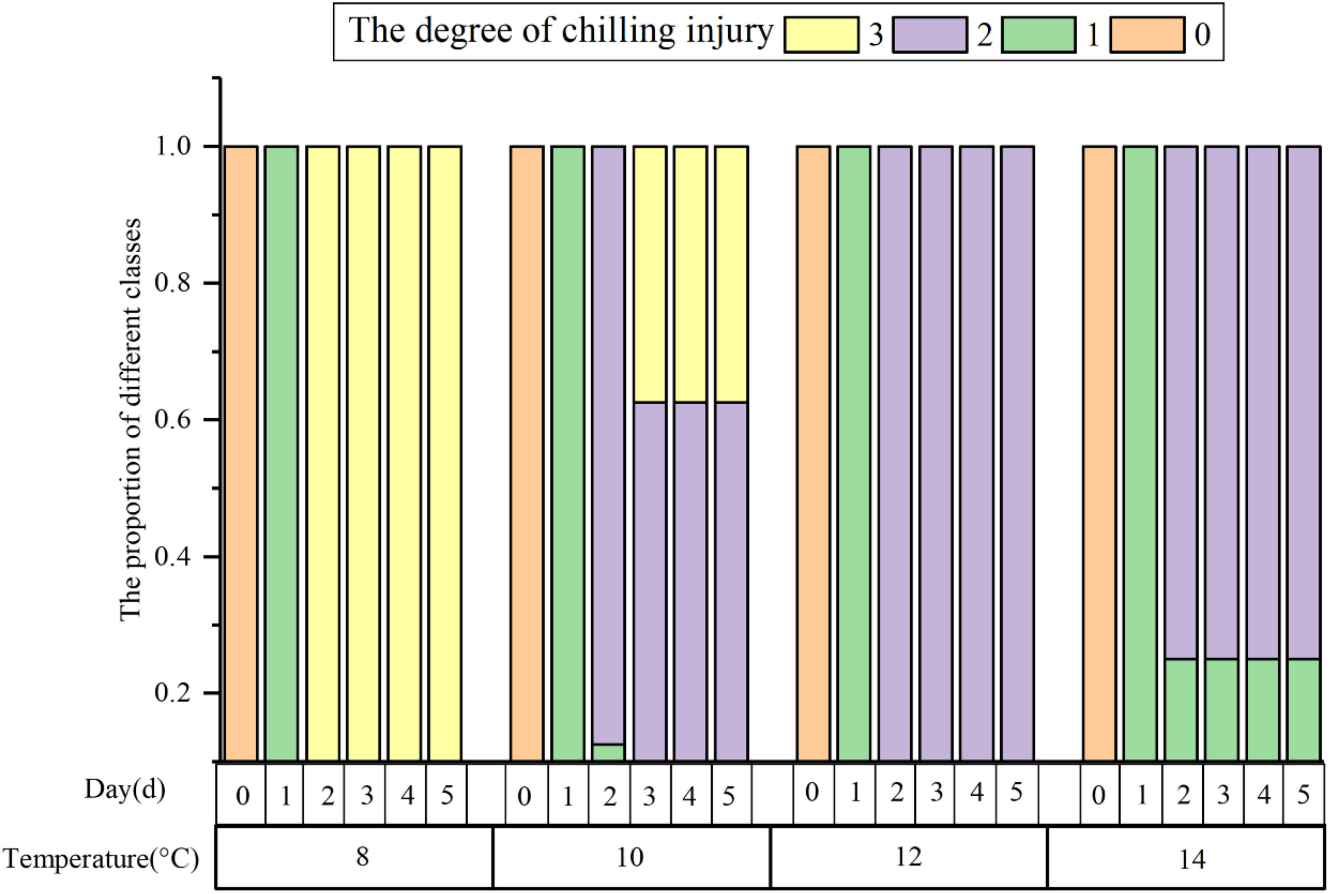
The relationship between the corresponding class of each sample and its environment.

All the samples were categorized into four chilling injury classes according to the classification model. **Figure 9** illustrates the severity of cucumber seedlings chilling injury under different low temperatures and time periods. The chilling injury levels of cucumber seedlings subjected to the same treatment may be different. After 1 d of low-temperature treatment, all samples were observed to have slight chilling injury. 25% of the samples treated at 14°C showed signs of slight chilling injury for 1 d or more. The samples treated at 8°C for 2 d and 37.5% of those treated at 10°C for 4 d were categorized as suffering from severe chilling injury. Under the other environmental treatments, the samples were categorized as having moderate chilling injury. Many studies have broken down the extent of chilling injury by temperature and duration, which may be applicable to the lengthening of time at a temperature or the lowering of temperature at a time, but this evaluation standard is constrained when both exist because of individual differences and subjective judgment. The classification model for evaluating cucumber chilling injury that uses a clustering algorithm is more objective and effectively reduces the influence of individual differences.

### 3.3 Cucumber seedlings under different chilling injury classes

#### 3.3.1 Changes in MDA content and ChlF images

**Figure 10:**
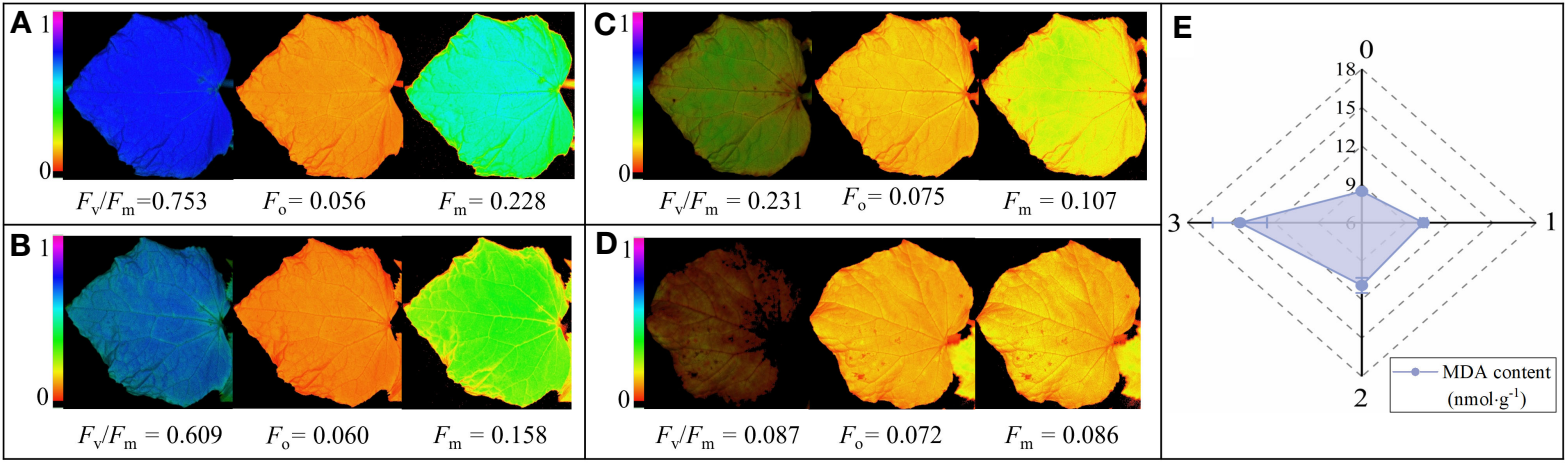
The MDA contents, *F*_v_/*F*_m_, *F*_m_, and *F*_o_ false-color images of cucumber seedlings under different chilling injury classes. **(A-D)** represent the *F*_v_/*F*_m_, *F*_m_, and *F*_o_ false-color images and their values of cucumber seedling under chilling injury class 0, 1, 2, and 3, respectively. **(E)** represents the MDA content of cucumber seedling under four chilling injury classes.

MDA contents and *F*_v_/*F*_m_, *F*_m_, and *F*_o_ false-color images of the samples with different chilling injury classes were analyzed (**Figure 10**). Low temperature stress causes changes in cell membrane permeability, transforming the membrane from a liquid crystalline state to a gel state, resulting in an increase in MDA content (Amin et al., 2021). Compared to unstressed samples, the average values of MDA contents with slight, moderate, and severe chilling injury samples increased 19.07%, 28.14%, and 67.24%, respectively (**Figure 10E**). The variations were minor at low chilling injury classes, indicating that the relationship between the MDA content and the degree of low temperature stress was nonlinear. Using MDA content to accurately evaluate the chilling injury classes requires the classification model proposed in this research. ChlF images are well suited to visualize spatio-temporal heterogeneity in plants’ responses under stress conditions (Fathi-Najafabadi et al., 2021). For *F*_m_ false-color images, they exhibited a color progression from cyan to green and eventually to yellow as the chilling injury intensified. With regard to the *F*_o_ false-color images, their color changes were not obvious under different chilling injury classes and different areas of cucumber leaves. The changes in *F*_v_/*F*_m_ were attributed to a decrease in *F*_m_ and an increase in *F*_o_. For *F*_v_/*F*_m_ false-color image, it is uniformly blue for the unstressed sample (**Figure 10A**); it appeared cyan when the cucumber seedling was affected by low-temperature (**Figure 10B**); it gradually became yellow-green, the main leaf veins appeared orange-red chilling spots with the deepening of chilling injury (**Figure 10C**); it turned to red, and some areas were identified as background areas because the *F*_v_/*F*_m_ values were less than 0.038 (**Figure 10D**). Spatial-temporal heterogeneity in the three parameters clearly illustrated the onset of cold sensitive symptoms in the vein and edge of leaves that progressed toward the center of leaves. Further research could be performed on the relationship between areas and classes of chilling injury using ChlF images. According to the above results, our model could be validated from MDA content and ChlF images.

#### 3.3.2 Changes in PSI and CEF

**Figure 11:**
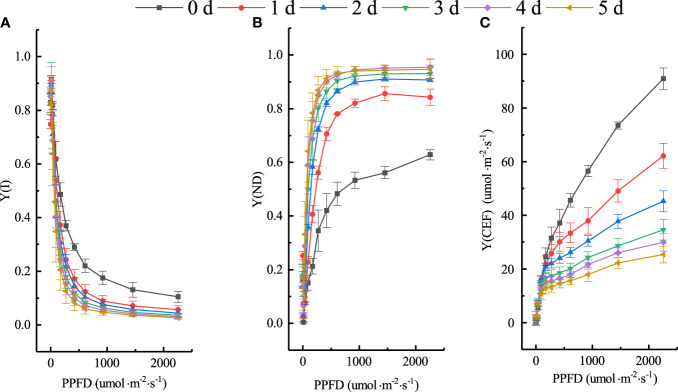
Changes of Y(I), Y(ND), and Y(CEF) under experimental conditions. **(A)** Y(I). **(B)** Y(ND). **(C)** Y(CEF).

The effect of low temperature on the photosynthetic reaction center of cucumber seedlings is not only reflected in PSII but also in PSI, which may be more cold-sensitive and not easily rectified. CEF is an important photoprotective mechanism that plays a crucial role in regulating the distribution of light energy between photosystems, balancing photoprotection, and facilitating the processes of photochemical reactions. Consequently, it is essential to investigate the changes in PSI and CEF under different chilling injury classes based on the classification model. We further analyzed the RLCs of Y(I), Y(ND), and Y(CEF). **Figure 11** depicts their distributions on different days at four low-temperature treatments. The parameter values of cucumber leaves remained stable at 0 d but changed rapidly when exposed to low temperatures. Under low-temperature treatment, the photosynthetic assimilation rate of cucumber seedlings decreased significantly and the photosynthetic electron transport chain became over-reduced, with captured light energy exceeding the absorption capacity of the electron transport chain. Excess light energy obstructed electron transfer, resulting in severe photoinhibition in both PSII and PSI. Thus, Y(I) and Y(CEF) decreased significantly. The high thermal dissipation capacity on the donor side [Y(ND)] was maintained to protect PSI from photoinhibition induced by strong light. Y(I) and Y(ND) changed linearly at low light intensity and reached a transition point at 420 to 610 μmol·m^-2^·s^-1^, and tended to stabilize at 921 μmol·m^-2^·s^-1^, indicating that the photoinhibition was deepened and Y(ND) was rapidly induced under strong light. Y(ND) differed substantially under different light intensities due to PSI’s limitation on accepting additional electrons for photosynthesis electron transfer on the donor side. Y(CEF) rose steadily with increasing light intensity and reached the maximum at 2250 μmol·m^-2^·s^-1^. In light of this, four light intensities of the RLCs (420, 610, 921, and 2250 μmol·m^-2^·s^-1^) were chosen to analyze the changes in PSI and CEF of cucumber seedlings under different chilling injury classes.

**Figure 12:**
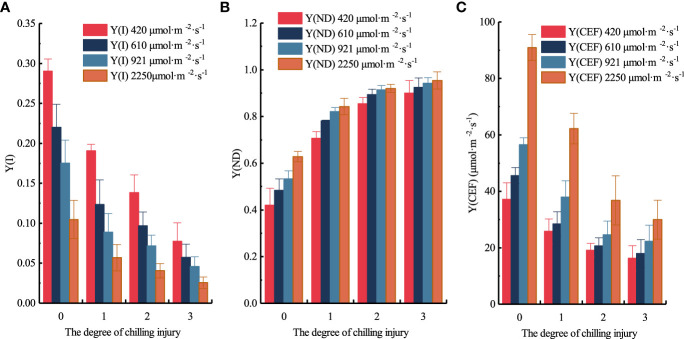
Distribution of Y(I), Y(ND), and Y(CEF) under different PAR and chilling injury classes. **(A)** Y(I). **(B)** Y(ND). **(C)** Y(CEF).

The distribution of Y(ND), Y(I), and Y(CEF) at each chilling injury class under the four light intensities are shown in **Figure 12**. The three parameters did vary between cucumber seedlings before and after low-temperature treatment, albeit some of them were not significant under different chilling injury classes. When the light intensity of the RLCs was 420 μmol·m^-2^·s^-1^, compared to unstressed samples, the Y(I) average values of slight, moderate, and severe chilling injury samples decreased by 34.350%, 52.413%, 73.403%, respectively; however, Y(ND) increased by 68.071%, 103.351%, 114.266%, respectively; and Y(CEF) decreased by 30.396%, 48.467%, 56.246%, respectively. When the light intensity of the RLCs was 610 μmol·m^-2^·s^-1^, compared to unstressed samples, the Y(I) average values of slight, moderate, and severe chilling injury samples decreased by 43.890%, 56.033%, 74.053%, respectively; Y(ND) increased by 61.601%, 84.931%, 91.302%, respectively; Y(CEF) decreased by 37.504%, 54.693%, 60.541%, respectively. when the light intensity of the RLCs was 921 μmol·m^-2^·s^-1^, compared to unstressed samples, the Y(I) average values of slight, moderate, and severe chilling injury samples decreased by 49.223%, 59.058%, 73.935%, respectively; Y(ND) increased by 54.021%, 71.605%, 76.924%, respectively; Y(CEF) decreased by 32.881%, 56.478%, 60.590%, respectively. when the light intensity of the RLCs was 2250 μmol·m^-2^·s^-1^, compared to unstressed samples, the Y(I) average values of slight, moderate, and severe chilling injury samples decreased by 45.708%, 61.155%, 75.435%, respectively; Y(ND) increased by 34.094%, 46.387%, 51.869%, respectively; Y(CEF) decreased by 31.599%, 59.590%, 67.052%, respectively. PSI regulates low temperature effects through high heat dissipation, thus, compared to unstressed samples, there is an obvious increase in Y(ND) in samples with slight chilling injury. The distribution of three parameters under different chilling injury classes indicated the validity of our classification model.

Since Y(CEF) is calculated by Y(I), Y(II), and light intensity, a positive correlation between Y(I) and Y(CEF) was consistently observed, and the relatively smaller decrease in Y(CEF) suggested that PSI is subjected to greater photoinhibition and damage compared to PSII. Furthermore, with the increase in chilling injury classes, the average value of Y(I) decreased by 43.293%, 57.165%, and 74.206%, respectively; the average value of Y(ND) increased by 54.447%, 76.569%, and 83.590%, respectively; the average value of Y(CEF) decreased by 33.094%, 54.807%, and 61.107%, respectively.

Low-temperature treatment could decrease the activity of PSI and induce PSI non-photochemical energy dissipation because of the donor-side limitation. When PSI is photo-inhibited, PSII is also more susceptible to photoinhibition due to the reduction in CEF, which aggravates the chilling injury and makes it difficult for plants to recover. The PSI photoinhibition of cucumber seedlings would be aggravated under strong light intensity or low-temperature conditions (Huang et al., 2016). As a result, the Y(I) values of cucumber seedlings with slight chilling injury and above were generally small. The Y(ND) values remained at a high level after low-temperature treatment, and there was no significant difference between moderate and severe chilling injury, demonstrating the donor side of PSI had a high thermal dissipation capacity (Lu et al., 2020). The rates of CEF in cucumber seedlings with moderate and severe chilling injury were largely inhibited, therefore, the Y(CEF) values were not significant. Because of this, PSI is more fragile than PSII and is more likely to be severely damaged when exposed to low temperature. In addition, PSII has a more efficient and dynamic recovery mechanism compared to PSI. Similarly, the PSI related parameters and the MDA contents could distinguish whether cucumber seedlings had been subjected to chilling injury to a certain extent, but they were not reliable in categorizing the chilling injury classes because their mechanisms of damage and recovery involve complex physiological reactions. We recommend that in future investigations, the PSII related parameters should be used to evaluate the chilling injury, and the PSI related parameters and biochemical parameters should be used to analyze the chilling injury.

## 4 Conclusion

Our study presents a novel approach for detecting chilling stress in cucumber seedlings by applying a classification model based on ChlF technology and the UMAP-GA-FCM algorithm. We investigated the relationship between ChlF parameters and the low temperature conditions experienced by cucumber seedlings. Through PCA, we identified some key ChlF parameters (*F*_v_/*F*_m_, Y(NO), qP, and *F*_o_) that were sensitive to chilling injury. Using the UMAP method, we extracted four new features (Feature 1, Feature 2, Feature 3, and Feature 4) from 15 ChlF parameters, which were then used as inputs for the classification model. Based on the UMAP-GA-FCM algorithm, the samples were categorized into four chilling injury classes: unstressed state (level 0), slight chilling injury (level 1), moderate chilling injury (level 2), and severe chilling injury (level 3). The distinct differences observed in ChlF images, PSI, CEF, and MDA contents among the different classes of cucumber seedlings validated the rationality of our proposed classification model. Our findings offer an expandable and non-destructive method for assessing plant responses to stress and the degree of damage incurred.

It should be noted that the experimental temperature range used in this study was already the lowest temperature that cucumbers could tolerate. Nonetheless, because there were a large number of samples and time-consuming experiment steps, the measurements were just once a day, making it impractical to classify the severity of chilling injury in a more fine-grained manner. In future research, we will consider enlarging the data set and employing automatic measurement techniques to obtain the chilling injury of cucumber seedlings at a more defined time node. Meanwhile, we will also consider changes in enzymes such as SOD, CAT, and POD under low temperature stress and the influence of temperature recovery on these parameters. We aim to incorporate more comprehensive data to enrich the classification model.

## Data availability statement

The raw data supporting the conclusions of this article will be made available by the authors, without undue reservation.

## Author contributions

ML: methodology, investigation, data collection and processing, writing—original draft, and writing—review and editing. JHou: data collection and writing—review and editing. PG: data collection and processing and writing—review and editing. DW: conceptualization, methodology, writing—review and editing. JHu: conceptualization, methodology, writing—review and editing, supervision, and project administration. All authors contributed to the article and approved the submitted version.
